# Z-peroral endoscopic myotomy made underwater to treat a short Zencker diverticulum with bad scope manoeuvrability

**DOI:** 10.1055/a-2868-6700

**Published:** 2026-06-08

**Authors:** Clément Juglard, Veronique Van der Voort, Mathieu Pioche, Romain Legros, Sophie Geyl, Jérémie Albouys, Jérémie Jacques

**Affiliations:** 1 Gastroentérologie et Endoscopie DigestiveCHU DupuytrenLimogesFrance; 2 Gastroenterology 1170 Meander Medisch CentrumAmersfoortThe Netherlands; 3 Unité d’Endoscopie Digestive, Service de GastroentérologieHôpital Edouard Herriot, Hospices Civils de LyonLyonFrance

Z-peroral endoscopic myotomy (Z-POEM) is increasingly used as the first-line endoscopic modality for the treatment of short Zenkerʼs diverticulum because of theoretical advantages in terms of recurrences. However, Z-POEM is technically more demanding than classic diverticulectomy in short diverticula because of very poor scope manoeuvrability in this location just below the piriform sinus 1 2 .

We present the case of a 76-year-old woman with a small Zenkerʼs diverticulum complicated by dysphagia, regurgitation and chronic cough. She was referred to our center for Z-POEM to incise the cricopharyngeal muscle down to the oesophageal muscle layer. In this case, using CO _2_ insufflation, the endoscope was very unstable and regularly ejected into the oropharynx.

The strategy adopted was to perform the procedure underwater in a patient protected by orotracheal intubation. Thanks to “water pressure” through the accessory channel throughout the procedure, the endoscope was stabilized without interfering with the conduction of the cutting current ( Video 1 ), allowing a quick and safe Z-POEM.

Underwater Z-peroral endoscopic myotomy to treat a short Zenkerʼs diverticulum.Video 1

**Fig. 1:**
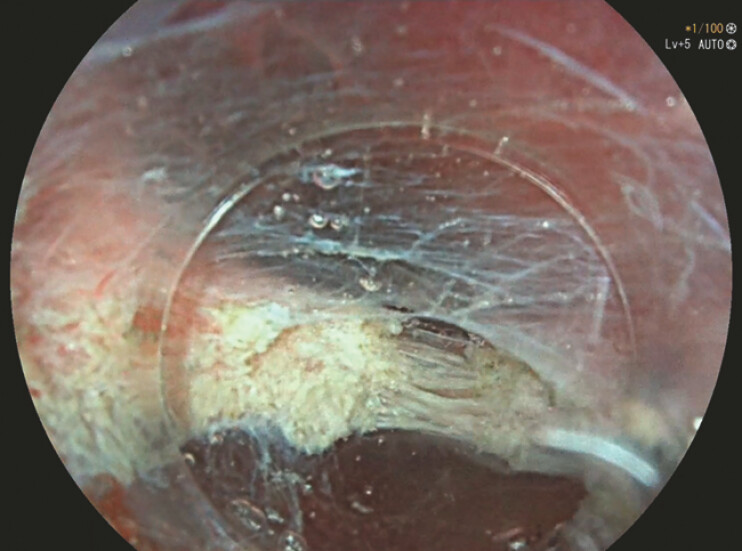
The two tunnels around the septum.

**Fig. 2:**
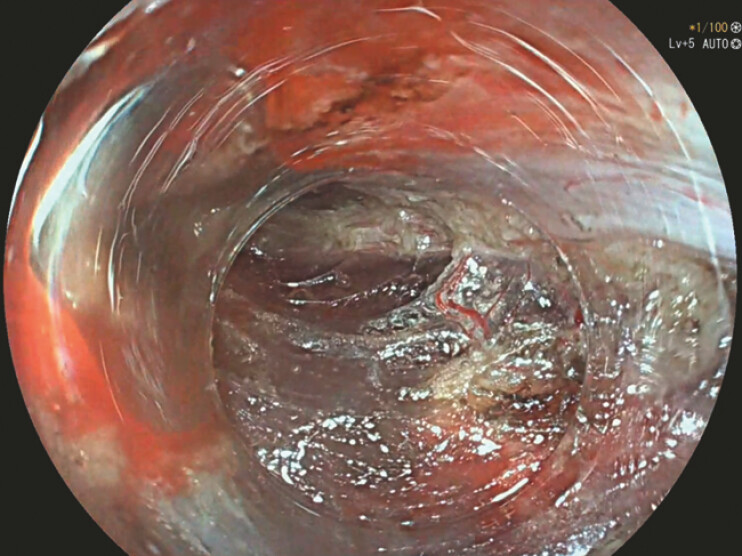
Complete septotomy.

The underwater procedure was similar to a standard procedure 3 . First, we inject the diverticular septum with a mixture of indigo carmine and glycerol. Then, we make a mucosal incision and two tunnels on either side of the septum ( Fig. 1 ). A complete septotomy was performed down to the oesophageal muscle ( Fig. 2 ). Visible bleeding was treated, and the tunnel was closed for safety reasons.

Underwater Z-POEM is an attractive solution for a short Zenkerʼs diverticulum to decrease the poor scope manoeuvrability that is often observed in this situation.

Endoscopy_UCTN_Code_TTT_1AO_2AP
